# Sublingual endothelial glycocalyx and atherosclerosis. A cross-sectional study

**DOI:** 10.1371/journal.pone.0213097

**Published:** 2019-03-27

**Authors:** Luca Valerio, Ron J. Peters, Aeilko H. Zwinderman, Sara-Joan Pinto-Sietsma

**Affiliations:** 1 Department of Public Health, Amsterdam UMC, University of Amsterdam, Amsterdam, Netherlands; 2 Department of Cardiology, Amsterdam UMC, University of Amsterdam, Amsterdam, Netherlands; 3 Department of Clinical Epidemiology and Biostatistics, Amsterdam UMC, University of Amsterdam, Amsterdam, Netherlands; 4 Department of Vascular Medicine, Amsterdam UMC, University of Amsterdam, Amsterdam, Netherlands; University Medical Center Utrecht, NETHERLANDS

## Abstract

**Background:**

Damage to endothelial glycocalyx is thought to be an early marker of atherosclerosis and measuring reduced glycocalyx size clinically via the Perfused Boundary Region (PBR) may allow early detection of cardiovascular disease. However, the true value of the glycocalyx in estimating cardiovascular risk or detecting cardiovascular disease is uncertain. We therefore investigated whether small glycocalyx size is associated with cardiovascular risk or disease in a large multi-ethnic cohort.

**Methods:**

In a multi-ethnic community-based sample (N = 6169, 42.4% male, mean age 43.6 ±13) we applied multiple imputation for missing data and used logistic regression and odds ratios to cross-sectionally investigate the relationship of small glycocalyx size as estimated by highest quartile of PBR with, on the one hand, classical risk factors for atherosclerosis including age, sex, diastolic and systolic blood pressure, LDL, HDL, triglycerides, BMI, diabetes, smoking status, and antihypertensive and lipid-lowering medication; on the other hand, prevalent cardiovascular disease. Analyses were additionally adjusted for ethnicity.

**Results:**

With PBR divided in quartiles, the highest PBR quartile (smallest glycocalyx size) as dependent variable was independently associated with female sex (OR for male versus female: 0.61, 95% CI: 0.53, 0.70) and diabetes (OR: 1.28, 95% CI: 1.03–1.59) in a model adjusted for all classical risk factors of atherosclerosis and for ethnicity. With regard to cardiovascular disease, no association was found between the smallest glycocalyx size as independent variable and overall cardiovascular disease, coronary heart disease and revascularization procedures, or stroke.

**Conclusions:**

Small glycocalyx size as estimated by highest PBR is associated with female sex and diabetes, which do not completely reflect a high cardiovascular risk profile. At the same time, glycocalyx size is not associated with prevalent cardiovascular disease.

## Introduction

The increasing burden of cardiovascular disease has led investigators to look for methods to identify atherosclerosis at an early stage. Evidence suggests that the earliest development towards atherosclerosis is structural and functional alteration of the vascular endothelium[1]. In particular, animal and human studies have shown that atherogenic stimuli and early atherogenesis are associated with structural and functional damage of the endothelial glycocalyx, a gel-like layer of membrane-attached carbohydrate polymers and adsorbed plasma proteins at the interface of vascular endothelial cells and flowing blood [2]. These associations have led to the hypothesis that detecting reduced glycocalyx size in a clinical setting may allow early detection of atherosclerosis [3].

Several methods have been developed to study the glycocalyx size in humans, but to date the only one suitable for clinical practice is the automatic calculation of the Perfused Boundary Region (PBR) from short video recordings of the sublingual microcirculation which measures lateral displacement of flowing erythrocytes and, when increased, possibly reflects reduced glycocalyx size and quality. Yet, its potential for use as a clinical risk estimation and prognostic tool is unclear [4]. We suggest that the increased PBR reflects damage to the vasculature, eventually resulting in atherosclerosis. Therefore, an increased PBR may be viewed as an intermediate step between risk factors for atherosclerosis and cardiovascular events [5–7]. However, the correlations between PBR and risk factors and between PBR and cardiovascular events have not been consistently shown for the PBR: some studies have reported no clear association between the PBR and either vascular risk or vascular disease [4, 7], while others have found associations with atherosclerotic vascular disease [6, 8, 9]. These inconsistent findings might depend on the relatively small sample size of these studies and the focus on populations selected based on health status and of predominantly white ethnicity [4–6, 10].

We therefore tested the hypothesis that a small glycocalyx as estimated by a high PBR is a marker of prevalent atherosclerosis and associated with the risk factors for atherosclerosis. We used this technology in a large-scale multi-ethnic population study, as a large sample size helps to overcome power limitations, while a multi-ethnic population displays a wide distribution of risk factors, thus making findings more generalizable.

## Patients and methods

### Study population

The HEalthy LIfe in Urban Settings (HELIUS) study is a cohort study on health among different ethnic groups living in Amsterdam, The Netherlands. The HELIUS study is described in detail elsewhere[11]. Briefly, baseline data collection took place in 2011–2015 among nearly 25,000 participants. Participants were randomly sampled from the municipal register, stratified by ethnicity, including people aged 18–70 years of Dutch origin or belonging to one of the ethnic minority groups with a Surinamese, Ghanaian, Turkish or Moroccan origin. Data were collected by questionnaire, physical examination and collection of biological samples. The study protocols were approved by the Ethical Review Board of the Academic Medical Centre of the University of Amsterdam (Protocol ID NL32251.018.10, approval number 10/100# 10.17.1729), and all individuals provided written informed consent.

PBR measurement was performed on a subsample of HELIUS participants, included between August 2012 and June 2014. Inclusion criteria for the current analysis were: attempted PBR measurement, successful blood draw, filled in questionnaire on lifestyle and medical history, and belonging to one of the ethnic groups studied in the HELIUS study. A total of 6169 participants fulfilled these criteria.

### Definitions

Ethnic origin was defined based on the country of birth of the participant and of his/her parents [12, 13]. Specifically, a participant is considered as of non-Dutch ethnic origin if he/she fulfils either of the following criteria: 1) he or she was born abroad and has at least one parent born abroad (first generation); or 2) he or she was born in the Netherlands but both his/her parents born abroad (second generation) [12]. The Surinamese group was further classified according to self-reported ethnic origin into ‘African’, ‘South Asian’, or ‘other/unknown’ Surinamese origin. Participants were considered of Dutch ethnicity if they and both parents were born in the Netherlands. Ethnicity was modelled as a categorical variables in six categories.

All participants were asked to bring their prescribed medications to the research location; these were identified and categorized by trained interviewers using the WHO Collaborating Centre for Drug Statistics Guidelines for Anatomical Therapeutic Chemical Classification [14].

Hypertension was defined as a systolic blood pressure ≥ 140 mmHg or a diastolic blood pressure ≥ 90 mmHg [15], or the combined report of prior diagnosis of hypertension *and* current use of antihypertensive agents (ATC codes C02, C03, C07, C08, C09) [14]. Blood pressure was measured using a validated automated digital BP device (Microlife WatchBP Home, Microlife AG, Heerbrugg, Switzerland) on the left arm in a seated position after the subject had been seated for at least 5 minutes.

Body mass index (BMI) was calculated as weight (kg), measured in light clothing with SECA 877 to the nearest 0.1 kg, divided by height squared (m^2^), with height measured without shoes with a portable stadiometer (SECA 217) to the nearest 0.1 cm. Waist circumference was measured to the nearest 0.01 m at the level midway between the lower rib margin and the iliac crest.

Blood samples were drawn in fasting state (at least 8 hours) and plasma samples were used to determine the concentration of LDL, HDL cholesterol, and triglycerides by colorimetric spectrophotometry (Roche Diagnostics, Japan). Diabetes was defined by fasting plasma glucose ≥ 7 mmol/l, with glucose concentration determined by spectrophotometry using hexokinase as primary enzyme (Roche Diagnostics, Japan), or the use of glucose-lowering medication (ATC code A10). Dyslipidemia was defined as either reported use of lipid-lowering agents (ATC code C10) or, using a common clinical risk estimator, cholesterol/HDL ratio ≥ 5 [16, 17]. Participants were considered smokers if they reported that they were currently smoking [18].

Cardiovascular disease diagnoses were based on questionnaire data. Patients with self-reported myocardial infarction, angioplasty in the coronary or peripheral circulation, or bypass surgery in either circulation were classified as having coronary heart disease or revascularization procedures. Stroke was also self-reported. Overall cardiovascular disease was defined as either of the above.

### Measurement of the PBR

In our study, image acquisition from participants occurred under standardized conditions: between 8:00 and 12:00, after at least eight hours of fasting and discontinuation of all medication, after a 60-minute physical examination, and in sitting position.

The measurement procedure and the calculation of the PBR have been described in detail [19, 20]. In short, image acquisition is semi-automated: the probe of a hand-held sidestream darkfield videomicroscope (MicroVision Medical Inc., Wallingford, PA), is placed on the sublingual mucosa of the participant until enough video frames of the sublingual microvasculature are obtained for the subsequent calculation; this recording takes approximately 2 minutes. Subsequently, an analysis software (GlycoCheck ICU, Glycocheck BV, Maastricht, the Netherlands) calculates the PBR by identifying vascular segments and estimating the dynamic lateral erythrocyte movement into the glycocalyx in μm, which estimates the accessibility of the glycocalyx to erythrocytes and, if increased, reflects a disturbance of the glycocalyx structure and function.

PBR calculation is fully automated and blinded to the investigator. Further details on the technique have been published previously [19] and an overview is provided in the online supplemental data (S2 Appendix).

To obtain the largest contrast in PBR and thus better identify its correlates, we used in subsequent analyses the highest PBR quartile versus the remaining three quartiles.

### Statistical analysis

We analysed the two associations expected of an early marker of atherosclerosis: first, the association of risk factors for atherosclerosis with highest PBR; second, the association of highest PBR with prevalent atherosclerotic cardiovascular disease.

The sample size consisted of 6169 individuals. Of these, 1209 (19.6%) had missing values in one or more variables, including the PBR measurement (S1 Appendix). In particular, 1031 subjects (16.7%) had a missing PBR because imaging was reported as unsuccessful by the measurement software, which was comparable with the 15% missing measurements reported in a previous cohort study [7]. In the other variables with missing values, the missing proportion was consistently very low, varying between 0.1% (1/6169) for BMI and 1.2% (72/6169) for self-reported coronary or peripheral vascular disease. All missing data for all variables that were planned for use in the subsequent analyses were imputed using multiple imputation according to the Chained Equations algorithm; 10 imputed datasets were generated. The analysis results on the single datasets were pooled using Rubin’s rules [21]. The PBR distribution in μm in the multiply imputed datasets as described by minimum, maximum, and quartiles, and reported in Table 1, was identical to its distribution in the complete cases dataset up to and including the second decimal figure (S1 Appendix).

**Table 1:** Sample characteristics by quartiles of Perfused Boundary Region (glycocalyx size).

		Quartiles of Perfused Boundary Region distribution				
		Large glycocalyx			Small glycocalyx	
		1^st^	2^nd^	3^rd^	4^th^	
N = 6169	Whole sample	[1.07 μm, 1.76 μm]	(1.76 μm, 1.93 μm]	(1.93 μm, 2.13 μm]	(2.13 μm, 3.11 μm]	P-value^a^
Male sex	2611 (42.3)	884 (57.3)	657 (42.6)	549 (35.6)	521 (33.8)	<0.001
Age, years	43.6 ±13.0	42.5 ±13.4	43.8 ±13.0	43.6 ±12.8	44.5 ±12.7	<0.001
Hypertension	1854 (30.1)	467 (30.3)	464 (30)	451 (29.3)	472 (30.6)	0.920
Use of antihypertensive agents	983 (15.9)	210 (13.6)	263 (17)	238 (15.5)	272 (17.6)	0.019
Blood pressure not on medication						
Systolic pressure, mmHg	125.4 ±16.4	127.0 ±16.0	124.8 ±15.9	124.7 ±16.6	124.9 ±16.9	<0.001
Diastolic pressure, mmHg	77.8 ±10.4	78.7 ±10.3	77.4 ±10.1	77.5 ±10.7	77.6 ±10.5	<0.001
BMI, kg/m^2^	27.3 ±5.3	27.1 ±5.1	27.4 ±5.3	27.5 ±5.4	27.3 ±5.4	0.196
Waist circumference, cm	92.6 ±13.3	92.9 ±13.0	92.7 ±13.4	92.7 ±13.5	92.1 ±13.1	0.271
Diabetes mellitus	565 (9.2)	118 (7.6)	145 (9.4)	145 (9.4)	157 (10.2)	0.135
Dyslipidemia	1382 (22.4)	368 (23.9)	358 (23.2)	340 (22.1)	316 (20.5)	0.238
Use of lipid-lowering agents	605 (9.8)	135 (8.7)	170 (11)	158 (10.2)	142 (9.2)	0.212
Blood lipids not on med.						
HDL, mmol/l	1.45 ±0.42	1.40 ±0.41	1.45 ±0.41	1.46 ±0.42	1.48 ±0.42	<0.001
Triglycerides, mmol/	0.98 ±0.68	1.03 ±0.81	0.99 ±0.65	0.96 ±0.63	0.93 ±0.61	<0.001
Current smoking	1359 (22)	367 (23.8)	353 (22.9)	331 (21.5)	308 (19.9)	0.146
Ethnicity						
Dutch	933 (15.1)	224 (14.5)	221 (14.3)	214 (13.9)	275 (17.8)	0.066
South-Asian Surinamese	1070 (17.3)	281 (18.2)	264 (17.1)	290 (18.8)	235 (15.2)	0.233
African Surinamese	793 (12.9)	155 (10.1)	185 (12)	180 (11.7)	272 (17.6)	<0.001
Ghanaian	938 (15.2)	224 (14.6)	233 (15.1)	215 (14)	266 (17.2)	0.135
Turkish	1194 (19.4)	316 (20.5)	291 (18.9)	325 (21.1)	262 (17)	0.087
Moroccan	1241 (20.1)	341 (22.1)	349 (22.6)	318 (20.6)	233 (15.1)	<0.001
Cardiovascular disease	319 (5.2)	77 (5)	82 (5.3)	75 (4.9)	84 (5.5)	0.931
Coronary disease / revascularizations	231 (3.7)	59 (3.9)	53 (3.4)	55 (3.6)	63 (4.1)	0.852
Stroke	106 (1.7)	24 (1.5)	35 (2.3)	21 (1.4)	26 (1.7)	0.339

Data are the average of 10 multiply imputed datasets. Summary statistics are presented by column. Continuous data are presented as mean ± SD; categorical data are presented as frequency (%).

^a^ pooled *p* value <0.05 versus glycocalyx size for chi-square trend test for trend (categorical data) or Spearman’s rho (continuous data).

Prior to the analysis, we studied the distribution of risk factors across quartiles of PBR by identifying monotonic associations between each risk factor and the PBR, based on the assumption that higher PBR reflects smallest glycocalyx size (see “Measurement of the PBR”). Categorical variables are presented as frequencies (%), normally distributed continuous variables as mean ± SD. To study their association with the PBR, we used the chi-square trend test for categorical risk factors and spearman’s rho for continuous ones.

Then, multivariate logistic regression and Odds Ratios (OR) were used to investigate the two associations of interest. To investigate whether small glycocalyx size was associated with risk factors for atherosclerosis, smallest glycocalyx size as defined by highest PBR quartile was used as dependent variable, and the following risk factors were used as independent variables in a first multivariate model: age, sex, systolic blood pressure, diastolic blood pressure, body mass index, blood LDL levels, blood HDL levels, blood triglyceride levels, diabetes, current smoking, use of antihypertensive agents, and use of lipid-lowering agents. All continuous variables were divided into quintiles for better comparison of their OR towards smallest glycocalyx size as defined by highest PBR quartile. A second model was additionally adjusted for ethnicity because the sample was multi-ethnic and because the above exploratory analysis showed the PBR to be distributed differently across ethnicities. If adjustment for ethnicity considerably affected the effect size of any variable present in the model, we verified whether the interaction between that variable and ethnicity was statistically significant.

To investigate whether small glycocalyx size was associated with atherosclerotic cardiovascular disease, three outcome measures were used as dependent variables: overall cardiovascular disease, coronary heart disease and revascularization procedures, and stroke. The latter two were considered as separate outcomes because, while they are both associated with atherosclerosis, they are known to be associated with different risk factor profiles. These associations were tested both in a univariate model in which smallest glycocalyx size as identified by highest PBR quartile was the only independent variable and, to identify a possible independent association of the PBR with prevalent atherosclerosis not explained by any association of the risk factors with the PBR but rather by any unmeasured determinants of the PBR, in a model additionally adjusted for all risk factors used in the first analysis.

All analyses were repeated on the complete cases dataset (S1 Appendix). Two-tailed *p* values of less than .05 were considered significant. Data were analysed using R 3.2.2 (The R Foundation for Statistical Computing, 2015).

## Results

### Sample characteristics

Participant characteristics are reported in Table 1. Mean age was 43.6 years ±13, and 42.4% of the participants were male. Cardiovascular disease was reported by 319 participants (5.2%), of which 231 (3.7%) reported coronary heart disease or revascularization procedures and 106 (1.7%) stroke.

To compare sample characteristics with PBR, the sample was stratified by quartile of PBR expressed in μm. Since the PBR is considered to be inversely associated with glycocalyx size, glycocalyx size is largest in the lowest quartile (left-hand column) and smallest in the highest quartile (right-hand column). In Table 1, a relationship with highest PBR could be observed for increasing age and use of antihypertensive agents. A similar although non-significant trend was observed for diabetes. An inverse relationship of a lower cardiovascular risk with small glycocalyx size could be observed for female sex, higher HDL cholesterol, lower triglyceride levels and lower systolic and diastolic blood pressures. A similar although non-significant trend was observed for non-smoking individuals.

### Association between PBR and cardiovascular risk factors

The association between cardiovascular risk factors and PBR as estimated by highest PBR is reported in Table 2. After adjusting for ethnicity, we observed that female sex, older age, higher diastolic blood pressure, lower BMI and diabetes together are best related to highest PBR. Of these associations, the only statistically significant ones were those with sex, age, and diabetes (OR for the association of male sex with highest PBR: 0.59, 95% CI: 0.50, 0.70; OR for the association of one quintile increase of age: 1.06, 95% CI: 1.00, 1.12; OR for the association of diabetes with highest PBR: 1.40, 95% CI: 1.10, 1.77, all *p*<0.05). Systolic blood pressure, smoking status, LDL levels, HDL levels and triglyceride levels were not retained in to the model. Introducing the interaction between ethnicity and diabetes did not change the results, and the association of interaction term with highest PBR was not statistically significant.

**Table 2:** Multivariate logistic regression analysis of the association between cardiovascular risk factors and highest PBR quartile (smallest glycocalyx size).

Outcome: highest PBR quartile		
Model	OR (95% CI)	P-value
Model 1		
Male sex	0.61 (0.52,0.71)	<0.001
Age (quintiles years)	1.09 (1.03,1.15)	0.002
Systolic blood pressure (quintiles mmHg)	0.95 (0.88,1.02)	0.152
Diastolic blood pressure (quintiles mmHg)	1.06 (0.99,1.14)	0.077
BMI (quintiles m/kg^2^)	0.95 (0.90,1.00)	0.068
LDL (quintiles mmol/L)	1.02 (0.97,1.07)	0.474
HDL (quintiles mmol/L)	0.99 (0.93,1.05)	0.670
Triglycerides (quintiles mmol/L)	0.97 (0.92,1.03)	0.289
Diabetes	1.31 (1.03,1.65)	0.027
Smoking status	0.94 (0.80,1.12)	0.498
Use of antihypertensive agents	1.12 (0.91,1.37)	0.276
Use of lipid-lowering agents	0.77 (0.59,0.99)	0.049
Model 2		
Male sex	0.59 (0.50,0.70)	<0.001
Age (quintiles years)	1.06 (1.00,1.12)	0.036
Systolic blood pressure (quintiles mmHg)	0.94 (0.87,1.01)	0.089
Diastolic blood pressure (quintiles mmHg)	1.03 (0.96,1.10)	0.411
BMI (quintiles m/kg^2^)	0.97 (0.92,1.03)	0.285
LDL (quintiles mmol/L)	1.01 (0.96,1.07)	0.602
HDL (quintiles mmol/L)	0.95 (0.90,1.01)	0.102
Triglycerides (quintiles mmol/L)	1.00 (0.95,1.06)	0.957
Diabetes	1.40 (1.10,1.77)	0.006
Smoking status	0.88 (0.74,1.05)	0.149
Use of antihypertensive agents	1.04 (0.84,1.28)	0.728
Use of lipid-lowering agents	0.82 (0.62,1.07)	0.141

In model 1 (multivariate): age quintiles (30, 41, 48, 56 years); systolic blood pressure quintiles (113.5, 121.5, 129.5, 141 mmHg); diastolic blood pressure quintiles (69.5, 75.5, 80.9, 87.5 mmHg); BMI quintiles (22.9, 25.4, 27.9, 31.4 kg/m^2^); LDL quintiles (2.2, 2.7, 3.2, 3.7 mmol/L); HDL quintiles (1.1, 1.3, 1.5, 1.8 mmol/L); triglycerides quintiles (0.5, 0.7, 1, 1.4 mmol/L).

Model 2 (multivariate): Model 1 + ethnicity (S1 Appendix).

### Association between PBR and vascular disease

The association between highest PBR and the atherosclerotic cardiovascular disease outcomes is reported in Table 3. Although the point estimate of the OR for the association between highest PBR and coronary heart disease and revascularization is above 1.0 both in the univariate and the multivariate model, this association was not statistically significant (univariate OR: 1.14, 95% CI: 0.83, 1.57; adjusted OR: 1.34, 95% CI: 0.95,1.91). The analysis on the outcomes overall cardiovascular disease and stroke also showed no association with highest PBR in either the univariate model, which tested the overall association of the PBR with the atherosclerotic outcomes, or the multivariate model, which tested the residual association of the PBR with the atherosclerotic outcomes independent of its association with the traditional risk factors.

**Table 3:** Univariate and multivariate logistic regression analysis of the association between highest PBR (smallest glycocalyx size) and cardiovascular disease outcomes.

Outcome:	Cardiovascular disease		Coronary heart disease and revascularization procedures		Stroke	
N events	319 (5.2%)		231 (3.7%)		106 (1.7%)	
	OR (95% CI)	*p*	OR (95% CI)	*p*	OR (95% CI)	*p*
Model 1						
Highest PBR quartile	1.08 (0.81,1.44)	0.608	1.14 (0.83,1.57)	0.406	0.95 (0.57,1.57)	0.840
Model 2						
Highest PBR quartile	1.19 (0.87,1.64)	0.281	1.34 (0.95,1.91)	0.098	0.92 (0.54,1.56)	0.759
Male sex	1.57 (1.17,2.10)	0.003	1.78 (1.26,2.50)	0.001	1.07 (0.67,1.72)	0.767
Age (quintiles years)	1.61 (1.42,1.83)	0.000	1.82 (1.56,2.13)	0.000	1.30 (1.06,1.58)	0.011
Systolic blood pressure (quintiles mmHg)	0.93 (0.82,1.07)	0.315	0.92 (0.79,1.07)	0.272	0.95 (0.77,1.18)	0.659
Diastolic blood pressure (quintiles mmHg)	0.91 (0.80,1.04)	0.159	0.89 (0.77,1.04)	0.134	1.00 (0.81,1.23)	0.987
BMI (quintiles m/kg^2^)	1.09 (0.98,1.22)	0.130	1.06 (0.93,1.21)	0.388	1.13 (0.95,1.34)	0.181
LDL (quintiles mmol/L)	0.86 (0.78,0.95)	0.004	0.87 (0.77,0.98)	0.018	0.87 (0.74,1.03)	0.106
HDL (quintiles mmol/L)	0.89 (0.80,1.00)	0.045	0.88 (0.77,1.00)	0.051	0.93 (0.78,1.11)	0.415
Triglycerides (quintiles mmol/L)	1.06 (0.95,1.19)	0.308	1.12 (0.98,1.28)	0.095	0.97 (0.81,1.16)	0.747
Diabetes	0.92 (0.66,1.29)	0.622	0.89 (0.61,1.30)	0.550	1.10 (0.64,1.89)	0.737
Smoking status	1.47 (1.10,1.97)	0.009	1.29 (0.92,1.82)	0.145	1.82 (1.16,2.85)	0.010
Use of antihypertensive agents	2.35 (1.73,3.20)	0.000	2.49 (1.75,3.55)	0.000	2.11 (1.27,3.51)	0.004
Use of lipid-lowering agents	2.93 (2.10,4.10)	0.000	2.69 (1.83,3.95)	0.000	2.40 (1.36,4.21)	0.002

Model 1: univariate. Model 2: multivariate; additionally adjusted for ethnicity (S1 Appendix).

## Discussion

We found that small glycocalyx size as defined by highest PBR was most strongly associated with older age, female sex, higher diastolic blood pressure, lower BMI, and diabetes. Of these associations, only those with sex and diabetes were statistically significant after correcting for possible confounders such as age, diastolic blood pressure, and BMI. These results are partly consistent with previous studies, which observed that diabetes was related to highest PBR [5, 10]. Both lower BMI and female sex have not been shown to be related to highest PBR: this is unexpected, since they are known to be inversely related to atherosclerotic disease. Although we observed slight ethnic differences, they did not confound the results. Finally, highest PBR was not associated with overall prevalent cardiovascular disease, coronary heart disease and revascularization procedures, or stroke.

Previous studies using the PBR to estimate glycocalyx size failed to show consistent associations between glycocalyx size and risk factors for atherosclerosis. In the study by Amraoui (2014) on 150 individuals, glycocalyx size was not associated with any vascular risk factor[4]. Gu et al. (2015) studied 726 individuals and standardized the PBR by haematocrit, pulse rate and perfused capillary density [7]. In this analysis, small glycocalyx size was associated with lower BMI, lower mean arterial pressure, and lower diastolic blood pressure, which is also in contrast with what would be expected concerning cardiovascular disease. The authors speculate that a higher vascular risk profile is associated with functional recruitment of capillaries with preserved glycocalyx. While our findings do not match those of this study, when the PBR is modelled as a continuous variable, increasing systolic blood pressure shows an independent association with lower PBR, all other results remaining unchanged (S1 Appendix). An association of PBR as a continuous variable with systolic blood pressure as well as markers of cardiac function has been reported in untreated hypertensives [22].

The observed association of highest PBR with age and to a lesser extent with female sex has not been reported previously. In particular, the association of highest PBR with female sex does not fit the hypothesis that a small glycocalyx size reflects higher cardiovascular risk. Others have suggested that glycocalyx size as estimated by the PBR should be standardized for haematocrit, since an association higher haematocrit and lower PBR has been observed that may in part explain the observed association with sex and age [4, 7].

The association between diabetes and highest PBR has been reported by Groen et al., in which a small sample of individuals with diabetes had a higher PBR than healthy controls[10]. This finding and ours are consistent with the existing hypotheses that a hyperglycaemic state reduces glycocalyx size[23, 24] and with a previous study that reported that glycocalyx size, estimated by a method similar to the PBR, was smaller among diabetes 2 patients than healthy controls[5]. Nevertheless, a small-scale observational study by Amraoui et al. was unable to show a relationship between PBR-estimated small glycocalyx size and diabetes[4], while a larger-scale observational study by Gu et al. did found no association between the Framingham risk score–that includes diabetes–or fasting blood glucose. These results could derive from insufficient power, or by the fact that in the study by Gu et al., the PBR was standardized by capillary density, which the authors report to be inversely associated with the PBR. Since diabetes has been reported to be associated with a decrease in capillary density [10, 25], this standardization may have obscured the association between diabetes and small glycocalyx size.

An association between PBR-estimated glycocalyx size and cardiovascular disease has been suggested by the observation that glycocalyx size is smaller in patients with premature atherosclerosis and their first-degree relatives compared to healthy controls [6], and by a recent report of an association of glycocalyx size with arterial wall lesions and history of both ischemic heart disease and cerebral disease in a selected sample [9]. However, most other studies in larger samples found no such associations for ischemic stroke [8] or history of vascular disease [4, 7]. Therefore, while glycocalyx size might be reduced in individuals with premature atherosclerosis and their families, this is not evident in subjects with vascular disease from the general population.

In combination with the findings of previous studies that investigated the PBR, our findings raise the question of whether the PBR is a viable indicator of glycocalyx size or quality. First, while animal studies and human studies conducted with other validated techniques did find associations between glycocalyx size and vascular risk or vascular disease [24, 26–28], most studies using the PBR have failed to do so. On the other hand, the best derivative of the PBR, the outward displacement of erythrocytes as an estimate of glycocalyx size, has been validated on animal in vivo studies, intravital microscopy, a commonly accepted gold standard method for the measurement of the glycocalyx size. In particular, the PBR was validated in a mouse study that showed how outward radial displacement of circulating RBCs significantly increased after experimental glycocalyx degradation via hyaluronidase [20]. The most important difference between the two measurements is that in the animal in vivo study the RBC-EC gap was visualized and represented the border of the endothelial wall, while in our method the PBR relies on an estimation of this position. The second question of whether the PBR is a viable indicator of the glycocalyx, follows from the fact that, even if the PBR is associated with the glycocalyx size, the resolution might be too low, or it might be significantly affected by other aspects of microvascular function. Indeed, the validation of the RBC-EC gap was conducted by experimentally degrading the glycocalyx, which might produce an impairment of its size and function significantly greater than those produced by the process of atherosclerosis in a relatively healthy human population studied observationally. Finally, one might question the reproducibility of the PBR measurement. This was investigated in another study by our research group [29] in which we found that the reproducibility of the PBR method for two single measurements in clinically homogeneous samples is poor, with an estimated Intraclass Correlation Coefficient lower than 0.50 for both intra-observer and inter-observer reproducibility, suggesting that a sample size in excess of 1,000 would be necessary to reach statistical significance for paired PBR differences. Based on this, our study has sufficient power, but the observed differences may not be biologically relevant.

An alternative interpretation of the inconsistency in the published findings is that, even if the PBR is accepted as a viable indicator of endothelial dysfunction, it only displays an association with vascular disease in selected samples of patients with advanced vascular or renal disease, but not in relatively healthy samples from the general population.

A number of limitations apply to this study. First, because the analysis is cross-sectional, we cannot infer that the associations are causal. Second, previous studies determined the PBR more than once per participant [7] or in several sublingual locations [4], while we only determined it once per participant and only in as many locations as necessary to obtain enough video frames for the PBR calculation. On the other hand, our PBR values are comparable to those reported in previous studies. Third, our outcomes of interest (coronary heart disease and stroke) were self-reported rather than based on medical records. This might have led to misclassification. On the other hand, questionnaires in HELIUS were completed with the help of a trained interviewer in a considerable proportion of the non-Dutch ethnicities, ranging from 27% of the Surinamese to 40% of the Ghanaians. We assume that any misclassification will only lead to a dilution of the effect, since self-reported cardiovascular disease in similar populations have been shown to have low sensitivity but high specificity, leading to false negatives rather than false positives [30, 31]. Fourth, in our study it was not possible to measure haematocrit and test its association with the PBR as done in previous studies.

Our study has several important strengths. First, this is the largest study that has investigated the association of the PBR with vascular risk factors and vascular disease. Second, we used a sound and comprehensive statistical method with multiple imputation for missing values to identify the combination of risk factors best representing a small glycocalyx as identified by highest PBR. Third, the multi-ethnic composition of the study sample resulted in a large spectrum of possible PBR, risk factors and cardiovascular diseases.

In conclusion, a PBR-estimated small glycocalyx size is cross-sectionally related to female sex and diabetes independent of ethnic background. There is no relationship between PBR-estimated glycocalyx size and prevalent cardiovascular disease.

## Supporting information

S1 Appendix Supplementary information. (DOCX)

S2 Appendix Parameters and measurement procedure. Supplementary information regarding the calculation of the SDF-derived microcirculation parameters Vascular Density, Red Blood Cell Filling, and Perfused Boundary Region and the measurement procedure in the HELIUS study.
(DOCX)
