# Supplementary material for: Sublingual endothelial glycocalyx and atherosclerosis. A cross-sectional study
Source: PLoS One. 2019 Mar 27;14(3):e0213097. doi: 10.1371/journal.pone.0213097 (PMC6436700; doi:10.1371/journal.pone.0213097)
Supplement: S1 Appendix — (DOCX) [file pone.0213097.s001.docx]

**S1 Appendix. Supplementary information.**

**Table S1. Effect size of ethnicity in Model 2 of the logistic regression of the association between risk factors for atherosclerosis and highest PBR.**

| **N = 6,169**  **Outcome: highest PBR quartile**  **(smallest glycocalyx size)** |  |  |
| --- | --- | --- |
| **Model** | **OR (95% CI)** | **P-value** |
| Model 2 |  |  |
| Ethnicity |  |  |
| Dutch (ref.) | - | - |
| South-Asian Surinamese | 0.65 (0.50,0.85) | 0.002 |
| African Surinamese | 1.23 (0.96,1.57) | 0.095 |
| Ghanaians | 0.93 (0.72,1.20) | 0.589 |
| Turks | 0.69 (0.54,0.88) | 0.002 |
| Moroccans | 0.51 (0.40,0.66) | 0.000 |

**Table S2. Effect size of ethnicity in Model 2 of the logistic regression of the association between highest PBR and cardiovascular disease outcomes.**

| **N = 6169**  **Outcome: highest PBR quartile**  **(smallest glycocalyx size)** | **Cardiovascular disease** | | **Coronary heart disease**  **and revascularization procedures** | | **Stroke** | |
| --- | --- | --- | --- | --- | --- | --- |
| **Model** | **OR (95% CI)** | **P-value** | **OR (95% CI)** | **P-value** | **OR (95% CI)** | **P-value** |
| Model 2 |  |  |  |  |  |  |
| Ethnicity |  |  |  |  |  |  |
| Dutch (ref.) | - | - |  |  |  |  |
| South-Asian Surinamese | 2.14 (1.32,3.47) | 0.002 | 2.85 (1.53,5.32) | 0.001 | 1.32 (0.66,2.63) | 0.437 |
| African Surinamese | 1.06 (0.60,1.86) | 0.851 | 1.12 (0.53,2.37) | 0.763 | 1.02 (0.48,2.20) | 0.957 |
| Ghanaians | 1.83 (1.06,3.15) | 0.029 | 2.62 (1.31,5.21) | 0.006 | 0.95 (0.42,2.14) | 0.906 |
| Turks | 2.18 (1.32,3.61) | 0.002 | 3.79 (2.01,7.16) | 0.000 | 0.70 (0.32,1.55) | 0.376 |
| Moroccans | 0.89 (0.51,1.57) | 0.692 | 1.56 (0.78,3.13) | 0.212 | 0.41 (0.16,1.02) | 0.056 |

**Table S3. Distribution of variables in patients with missing and not missing Perfused Boundary Region in the original data.**

| N=6,169 | PBR not missing | PBR missing |
| --- | --- | --- |
| col. %s | N=5,138 (83.3%) | N=1,031 (16.7%) |
| Male, n (%): | 2145 (41.7) | 466 (45.2)* |
| Age, years | 43.5 ±13.0 | 44.3 ± 13.0* |
| Hypertension, n (%): | 1545 (30.2) | 303 (29.5) |
| Systolic blood pressure, mmHg | 127.9 ±17.5 | 127.8 ±17.6 |
| Diastolic blood pressure, mmHg | 78.9 ±10.7 | 78.9 ±10.5 |
| On antihypertensive agents, n (%): | 807 (15.7) | 176 (17.1) |
| Blood pressure in those treated: |  |  |
| Systolic blood pressure, mmHg | 140.9 ±17.6 | 140.5 ±17.7 |
| Diastolic blood pressure, mmHg | 84.3 ±10.5 | 85.2 ±10.3* |
| Blood pressure in those untreated: |  |  |
| Systolic blood pressure, mmHg | 125.4 ±16.4 | 125.2 ±16.4 |
| Diastolic blood pressure, mmHg | 77.9 ±10.5 | 77.6 ±10.1* |
| BMI, m/kg^2^ | 27.4 ±5.3 | 26.9 ±5.2* |
| Waist circumference, cm | 92.8 ±13.4 | 91.7 ±12.7* |
| Diabetes, n (%): | 470 (9.1) | 95 (9.2) |
| Dyslipidemia, n (%): | 1157 (22.5) | 225 (21.8) |
| LDL, mmol/L | 3.00 ±0.89 | 3.01 ±0.93 |
| HDL, mmol/L | 1.43 ±0.41 | 1.44 ±0.44 |
| On lipid-lowering agents, n (%): | 507 (9.9) | 98 (9.5) |
| Blood lipids in those treated: |  |  |
| LDL, mmol/L | 2.49 ±0.90 | 2.46 ±0.88 |
| HDL, mmol/L | 1.28 ±0.37 | 1.41 ±0.56* |
| Triglycerides, mmol/L | 1.30 ±0.86 | 1.18 ±0.72* |
| Blood lipids in those untreated: |  |  |
| LDL, mmol/L | 3.05 ±0.88 | 3.06 ±0.91 |
| HDL, mmol/L | 1.45 ±0.41 | 1.44 ±0.42 |
| Triglycerides, mmol/L | 0.97 ±0.68 | 0.99 ±0.68* |
| Smoker, n (%) | 1074 (21) | 281 (27.3)* |
| Ethnicity |  |  |
| Dutch, n (%) | 736 (14.3) | 197 (19.1)* |
| South-Asian Surinamese, n (%) | 904 (17.6) | 166 (16.1) |
| African Surinamese, n (%) | 658 (12.8) | 135 (13.1) |
| Ghanaians, n (%) | 773 (15) | 165 (16) |
| Turks, n (%) | 980 (19.1) | 214 (20.8) |
| Moroccans, n (%) | 1087 (21.2) | 154 (14.9)* |
| CVD, n (%) | 265 (5.2) | 51 (5.1) |
| Coronary or peripheral vascular disease, n (%) | 194 (3.8) | 35 (3.5) |
| Stroke, n (%) | 89 (1.7) | 16 (1.6) |

Continuous data are presented as mean ± SD; categorical data are presented as frequency (%).

*Statistically significant difference for p < 0.05 for a t-test for continuous variables and a chi-square test for categorical variables.

**Figure S1. Distribution of the PBR in the subjects with non-missing PBR in the original data and in the 10 imputed datasets.**

**
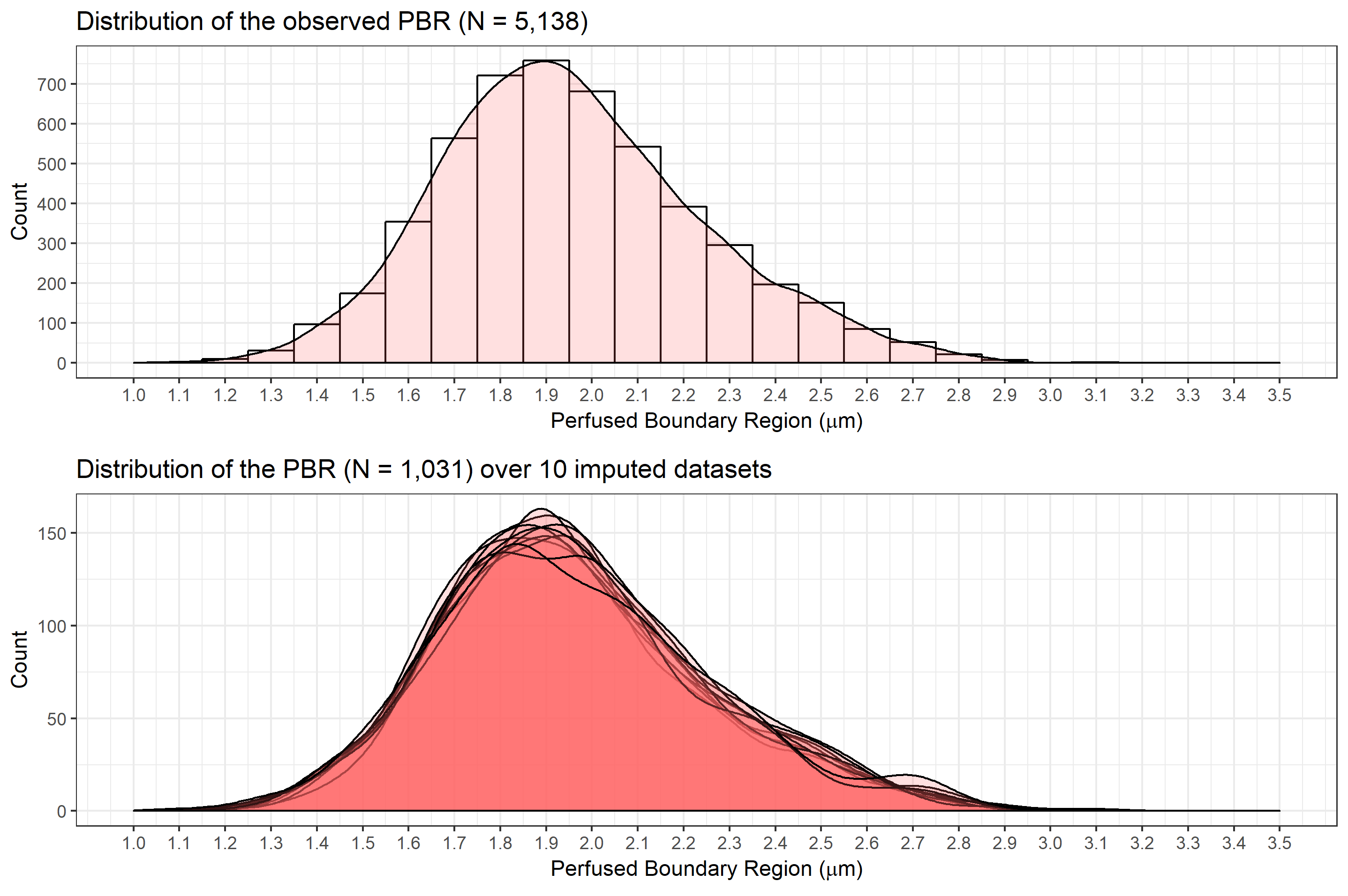
**

**Table S4. Logistic regression** **for the association between risk factors for atherosclerosis and highest PBR in the complete case analysis dataset (N = 4,960).**

| **Outcome: highest PBR quartile (N = 1, 240)** |  |  |
| --- | --- | --- |
| **Model 1** | **OR (95% CI)** | **P-value** |
| Male sex | 0.64 (0.54-0.74) | 0.000 |
| Age (higher quartile, years) | 1.09 (1.03-1.15) | 0.003 |
| Systolic blood pressure (higher quartile, mmHg) | 0.94 (0.87-1.01) | 0.096 |
| Diastolic blood pressure (higher quartile, mmHg) | 1.07 (1-1.15) | 0.048 |
| BMI (higher quartile, kg/m^2^) | 0.96 (0.91-1.01) | 0.129 |
| LDL (higher quartile, mmol/L) | 1.02 (0.97-1.08) | 0.381 |
| HDL (higher quartile, mmol/L) | 0.98 (0.92-1.03) | 0.391 |
| Triglycerides (higher quartile, mmol/L) | 0.95 (0.89-1) | 0.056 |
| Diabetes | 1.29 (0.99-1.68) | 0.054 |
| Smoker | 0.97 (0.82-1.15) | 0.742 |
| On antihypertensive agents | 1.12 (0.91-1.38) | 0.280 |
| On lipid-lowering agents | 0.78 (0.59-1.02) | 0.074 |
| **Model 2** |  |  |
| Male sex | 0.62 (0.53-0.73) | 0.000 |
| Age (higher quartile, years) | 1.06 (1-1.12) | 0.046 |
| Systolic blood pressure (higher quartile, mmHg) | 0.93 (0.86-1.00) | 0.042 |
| Diastolic blood pressure (higher quartile, mmHg) | 1.04 (0.97-1.12) | 0.302 |
| BMI (higher quartile, kg/m^2^) | 0.98 (0.93-1.04) | 0.510 |
| LDL (higher quartile, mmol/L) | 1.02 (0.97-1.07) | 0.492 |
| HDL (higher quartile, mmol/L) | 0.94 (0.88-0.99) | 0.029 |
| Triglycerides (higher quartile, mmol/L) | 0.98 (0.92-1.04) | 0.514 |
| Diabetes | 1.41 (1.08-1.83) | 0.011 |
| Smoker | 0.9 (0.76-1.07) | 0.252 |
| On antihypertensive agents | 1.03 (0.83-1.27) | 0.782 |
| On lipid-lowering agents | 0.83 (0.63-1.10) | 0.208 |
| Ethnicity |  |  |
| Dutch (ref.) | - | - |
| South-Asian Surinamese | 0.61 (0.48,0.77) | 0.000 |
| African Surinamese | 1.21 (0.95,1.53) | 0.126 |
| Ghanaians | 0.92 (0.72,1.18) | 0.519 |
| Turks | 0.64 (0.50,0.81) | 0.000 |
| Moroccans | 0.49 (0.39,0.62) | 0.000 |

In model 1 (multivariate): age quintiles (30, 41, 48, 55 years); systolic blood pressure quintiles (113.5, 121.5, 129.5, 141 mmHg); diastolic blood pressure quintiles (69.5, 75.5, 80.5, 87.5 mmHg); BMI quintiles (22.9, 25.5, 28.0, 31.4 kg/m^2^); LDL quintiles (2.2, 2.7, 3.2, 3.7 mmol/L); HDL quintiles (1.1, 1.3, 1.5, 1.8 mmol/L); triglycerides quintiles (0.5, 0.7, 1.0, 1.3 mmol/L).

Model 2 (multivariate): Model 1 + ethnicity.

**Table S5. Logistic regression on the association between** **PBR and cardiovascular disease in the complete case analysis dataset (N = 4,960)**

| Outcome: | Cardiovascular disease | | Coronary heart disease  and revascularization procedures | | Stroke | |
| --- | --- | --- | --- | --- | --- | --- |
| N events | 254 (5.1%) |  | 186 (3.8%) |  | 85 (1.7%) |  |
|  | OR (95% CI) | p | OR (95% CI) | p | OR (95% CI) | p |
| Model 1 |  |  |  |  |  |  |
| Highest PBR quartile | 1.03 (0.77-1.37) | 0.823 | 1.14 (0.81-1.57) | 0.438 | 0.86 (0.5-1.41) | 0.570 |
| Model 2 |  |  |  |  |  |  |
| Highest PBR quartile | 1.15 (0.83-1.58) | 0.385 | 1.37 (0.95-1.95) | 0.091 | 0.83 (0.48-1.4) | 0.507 |
| Male sex | 1.76 (1.27-2.44) | 0.001 | 1.95 (1.34-2.87) | 0.001 | 1.18 (0.7-1.99) | 0.527 |
| Age (quintiles years) | 1.66 (1.45-1.92) | 0.000 | 1.84 (1.55-2.19) | 0.000 | 1.4 (1.13-1.75) | 0.002 |
| Systolic blood pr. (quintiles mmHg) | 0.93 (0.8-1.08) | 0.344 | 0.95 (0.8-1.13) | 0.577 | 0.89 (0.7-1.13) | 0.341 |
| Diastolic blood pr. (quintiles mmHg) | 0.9 (0.78-1.04) | 0.171 | 0.86 (0.73-1.01) | 0.063 | 1.04 (0.83-1.31) | 0.754 |
| BMI (quintiles m/kg2) | 0.85 (0.76-0.95) | 0.006 | 0.87 (0.76-0.99) | 0.034 | 0.82 (0.69-0.99) | 0.037 |
| LDL (quintiles mmol/L) | 0.83 (0.73-0.94) | 0.004 | 0.85 (0.73-0.98) | 0.025 | 0.83 (0.67-1.01) | 0.061 |
| HDL (quintiles mmol/L) | 1.04 (0.92-1.18) | 0.529 | 1.1 (0.95-1.28) | 0.197 | 0.96 (0.79-1.18) | 0.691 |
| Triglycerides (quintiles mmol/L) | 1.04 (0.92-1.18) | 0.520 | 1.01 (0.87-1.16) | 0.945 | 1.09 (0.9-1.32) | 0.395 |
| Diabetes | 0.89 (0.61-1.3) | 0.546 | 0.91 (0.6-1.39) | 0.678 | 0.88 (0.47-1.61) | 0.674 |
| Smoking status | 1.29 (0.92-1.79) | 0.135 | 1.2 (0.81-1.76) | 0.357 | 1.41 (0.83-2.32) | 0.192 |
| Use of antihypertensive agents | 2.71 (1.92-3.83) | 0.000 | 3.07 (2.07-4.56) | 0.000 | 2.11 (1.19-3.75) | 0.011 |
| Use of lipid-lowering agents | 2.52 (1.73-3.68) | 0.000 | 2.22 (1.44-3.42) | 0.000 | 2.31 (1.22-4.35) | 0.010 |
| Ethnicity |  |  |  |  |  |  |
| Dutch (ref.) | - | - |  |  |  |  |
| South-Asian Surinamese | 1.86 (1.11-3.22) | 0.022 | 2.94 (1.48-6.4) | 0.004 | 1.03 (0.51-2.16) | 0.933 |
| African Surinamese | 0.91 (0.49-1.71) | 0.772 | 1.15 (0.5-2.79) | 0.743 | 0.82 (0.37-1.85) | 0.629 |
| Ghanaians | 1.35 (0.74-2.5) | 0.334 | 2.56 (1.19-5.92) | 0.020 | 0.49 (0.18-1.24) | 0.138 |
| Turks | 1.89 (1.1-3.34) | 0.024 | 4.12 (2.04-9.08) | 0.000 | 0.49 (0.21-1.15) | 0.099 |
| Moroccans | 0.71 (0.38-1.34) | 0.288 | 1.54 (0.71-3.57) | 0.292 | 0.25 (0.08-0.66) | 0.007 |

Model 1: univariate. Model 2: multivariate. Age quintiles (30, 41, 48, 55 years); systolic blood pressure quintiles (113.5, 121.5, 129.5, 141 mmHg); diastolic blood pressure quintiles (69.5, 75.5, 80.5, 87.5 mmHg); BMI quintiles (22.9, 25.5, 28.0, 31.4 kg/m^2^); LDL quintiles (2.2, 2.7, 3.2, 3.7 mmol/L); HDL quintiles (1.1, 1.3, 1.5, 1.8 mmol/L); triglycerides quintiles (0.5, 0.7, 1.0, 1.3 mmol/L).
